# The impact of antiretroviral therapy on HPV and cervical intraepithelial neoplasia: current evidence and directions for future research

**DOI:** 10.1186/1750-9378-5-8

**Published:** 2010-05-12

**Authors:** Lara F Bratcher, Vikrant V Sahasrabuddhe

**Affiliations:** 1 Institute for Global Health, Vanderbilt University School of Medicine, Nashville, USA; 2 Institute for Global Health and Department of Pediatrics, Vanderbilt University School of Medicine, Nashville, USA

## Abstract

Increasing numbers of human immunodeficiency virus (HIV)-infected women are now accessing life-prolonging highly active antiretroviral therapy (HAART) in developing countries. There is a need for better understanding of interactions of human papillomavirus (HPV) and HIV, especially in the context of increasing life expectancy due to HAART. The data regarding the impact of HAART on reducing the incidence and progression and facilitating the regression of HPV infection and cervical abnormalities is largely inconsistent. Published studies differ in their study designs (prospective or retrospective cohorts or record linkage studies), screening and diagnostic protocols, duration and type of HAART use, recruitment and referral strategies, and definitions of screening test and disease positivity. Due to the ethical and resource limitations in conducting randomized trials of the impact of HAART on incidence of HPV, CIN, and cervical cancer among HIV-infected women, it is important to consider innovative study designs, including quasi-experimental trials and operations research in sentinel populations to answer the critical research questions in this area.

## Background

Invasive cervical cancer (ICC), although entirely preventable by early detection and treatment, remains one of the most common malignancies among women worldwide[1]. ICC and its precursor, cervical intraepithelial neoplasia (CIN), are associated with persistent infection with oncogenic 'high-risk' (HR) types of the human papillomavirus (HPV). Products of HPV oncogenes E6 and E7 alter normal genetic and cellular functions and induce malignant transformation. In women with healthy immune systems, most HPV infections are cleared. However, for those with persistent HR-HPV infection, gradual accumulation of altered cellular genetic material may progress, leading to development of CIN (grades 1, 2, 3) and eventually ICC. These changes tend to originate at the squamocolumnar junction of the cervix and may increasingly involve surrounding cells. Characteristic changes in cervical cells throughout this progression can be detected at the pre-clinical stage, allowing for treatment of precursor lesions long before ICC occurs.

**Figure 1:**
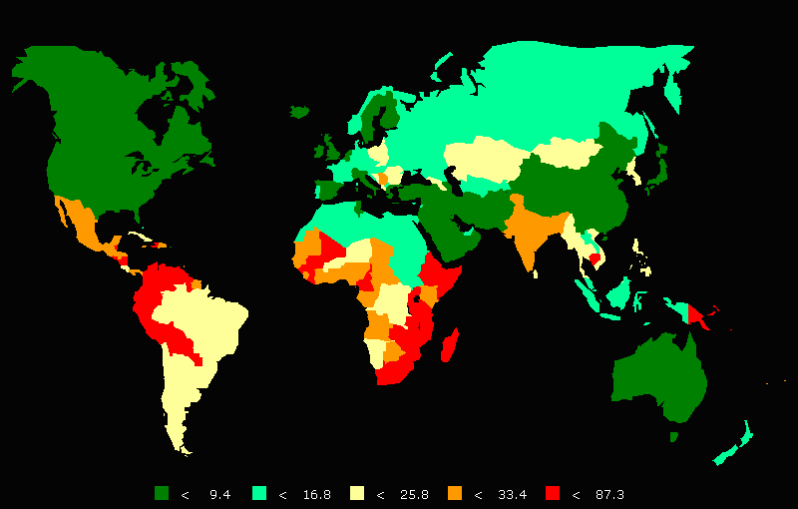
**Global cervical cancer age-standardized incidence rate per 100,000 women (2002) [Source: International Agency for Research on Cancer: Cancer Mondial**: http://www-dep.iarc.fr**]**.

**Figure 2:**
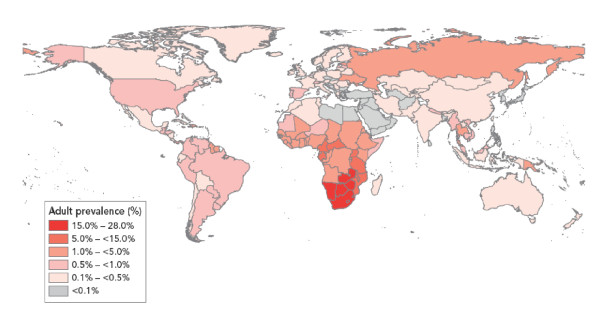
**Global HIV prevalence (2007) [Source: UNAIDS **http://www.unaids.org**]**.

The developing world carries the largest burden (over 80%) of ICC, largely as a result of the expense and logistical challenges in establishing and sustaining population-level screening and treatment programs. (Figure 1) In many resource-limited areas, it is the most common cause of death from malignancies in women[1]. Those same areas also carry a large burden of human immunodeficiency virus (HIV) and acquired immunodeficiency syndrome (AIDS) (Figure 2). HIV-infected women face an increased risk for the incidence,[2,3] persistence,[4,5] and recurrence [6,7] of HPV-induced anogenital and cervical neoplastic disease[8]. Although ICC has been labeled an AIDS-defining malignancy, there is still a limited understanding of the natural history and epidemiology of HPV-induced neoplastic disease in HIV-infected women.

The introduction of highly active antiretroviral therapy (HAART*) in the late nineties resulted in dramatic improvement of clinical outcomes and life expectancies for people living with HIV/AIDS. It also gave hope that improved immunological status would result in better clearance of HPV infection in HIV-infected women, much like other opportunistic and AIDS-associated infections, and result in a gradual decrease in the incidence and progression of cervical neoplasia. However, data from the industrialized world does not point to a clear reduction in the burden and severity of cervical disease with introduction of HAART, in contrast to other AIDS-related malignancies, most notably Kaposi's sarcoma and non-Hodgkin lymphoma [9-12].

The need for better understanding of the interactions between HIV and HPV in the context of HAART is therefore even more pressing as increasing numbers of HIV-infected women are living longer with a persistent risk of ICC. Guidelines for prevention and treatment strategies for cervical cancer among HIV-infected women are largely based on limited evidence, or in the case of resource limited settings, are completely lacking. In this review, we summarize the available epidemiological evidence in this area, nearly all of which originates from industrialized nations. We discuss the priorities for further research in relation to resource-limited settings, home to over 90% of women living with HIV/AIDS.

## Methods

A systematic review of literature documenting the impact of HAART on development of HPV-induced cervical intraepithelial neoplasia (CIN) in HIV-infected women was conducted using PubMed. Article selection criteria included any clinic-based observational or population-based linkage studies documenting both HAART status and HPV/CIN/ICC rates. PubMed was searched with an end date of January 2009 using Medical Subject Headings (MeSH) "Uterine Cervical Neoplasms" OR "AlphaPapillomavirus" AND "Antiretroviral Therapy, Highly Active" limiting to English language literature. A total of 59 articles retrieved through this search were reviewed manually and additional articles were retrieved by cross referencing. The final analysis included 22 papers that are tabulated in Table 1. We did not include studies that were not published as full manuscripts in peer-reviewed literature (e.g. conference abstracts or proceedings) or any unpublished or gray literature (e.g. project reports) to ensure inclusion of studies with completed (not interim) analyses.

**Table 1:** Description of included studies

Author	Design	No. of women/cases	Country	HAART Definition
Heard et al 1998	Prospective Cohort	533	France	2 NRTI + 1 PI
Heard et al 2002	Prospective cohort	168	France	2 NRTIs + 1 PI or 1 NNRTI
Heard et al 2006	Prospective cohort	289	France	as defined by French National Recommendations
Orlando et al 1999	Prospective cohort	15	USA	Undefined
Moore et al 2002	Prospective cohort	71	UK	Undefined
Dorucci et al 2001	Prospective cohort	6	Italy	NA
Lillo et al 2001	Prospective cohort	168	Italy	Undefined
Del Mistro et al 2004	Prospective cohort	201	Italy	1 PI, 2 NRTIs + NNRTI; or 3 NRTIs
Soncini et al 2007	Prospective cohort	101	Italy	2 NRTIs + 1 PI, or NNRTI; or 3 NRTIs
Sirera et al 2007	Retrospective cohort	133	Spain	2 NRTIs + 1 PI or 1 NNRTI
Sirera et al 2008	Retrospective cohort	127	Spain	2 NRTIs + 1 PI or 1 NNRTI
Ellerbrock et al 2000	Prospective cohort	328	USA	Various NRTI and NNRTI (1 or more in combination) defined by 1997 NIH guidelines or >2 PI multiple regimens defined
Minkoff et al 2001	Prospective cohort, WIHS	781	USA	
Ahdieh-Grant et al 2004	Prospective cohort, WIHS	312	USA	
Schuman et al 2003	Prospective cohort, HERS	774	USA	as defined by DHHS Guidelines
Paramsothy et al 2009	Prospective cohort, HERS	537	USA	as defined by DHHS Guidelines
Intl Collaboration on HIV and Cancer 2000	Meta-analysis of prospective studies evaluating cancer risk in HIV-infected persons	36	USA, Europe, Australia	NA
Clifford et al 2005	Prospective cohort	6	Switzerland	NA
Engels et al 2006	HIV/AIDS Cancer Match Study	64	USA	NA
Biggar et al 2007	HIV/AIDS Cancer Match Study	55	USA	NA
Engels et al 2008	HIV/AIDS Cancer Match Study	28	USA	NA
Dal Maso et al 2009	HIV/AIDS Cancer Match Study	39	Italy	NA

## Results

The published literature on this subject is striking in lack of standardization in study designs and methods of screening as well as the dearth of studies among populations from resource-limited settings. The differences in clinical versus population-based study designs, use of prevalent versus incident lesions as endpoints, different thresholds of diagnostic criteria for CIN, differing standards of lengths of follow-up evaluation periods, and nonuniform use of diagnostic assessments by colposcopy and/or histopathology make it difficult to draw overall conclusions about the impact of HAART on cervical disease. We discuss these differences by stage of HPV infection/cervical disease and highlight trends and similarities, as well as differences, in conclusions.

The definition of HAART also varies across studies, reflecting the evolution in terminology as well as the time horizon of adoption of newer classes of drugs over the past decade and a half. Several authors have defined HAART conventionally, i.e. as consisting of 2 nucleoside reverse transcriptase inhibitors (NRTIs) + 1 protease inhibitor (PI) or 1 non-nucleoside reverse transcriptase inhibitor (NNRTI) [13-15]. Some referred to definitions of HAART prevalent at their institutions, [16-19] while others have listed multiple definitions in their work[13,14,20,21]. Since viral suppression and immune reconstitution appears to be dependent on the class of drug and duration of treatment, this is an important source of variation to be considered before drawing broad conclusions. Furthermore, in several studies, HAART use was measured by self-report. Some studies compared patient groups using HAART to groups using unspecified combinations of antiretroviral drugs [17,20,22,23] while other studies have used antiretroviral-naïve patients in comparison groups[14,15]. CD4+ cell count has been uniformly used as a surrogate of immune status, with most studies controlling for CD4+ counts in the analysis[15,17,20,23,24]. Lower CD4+ cell counts have been shown to independently predict both incidence and progression of lesions,[25] and evidence of a dose-dependent relationship between CD4+ counts and regression of lesions has also been documented for women on HAART[13].

There are significant differences between studies in the utilization of the screening/diagnostic methods for cervical pathology. Most investigators have used cytological (Pap smear) changes with reporting based on the revised Bethesda classification, relying on low and high-grade squamous intraepithelial lesions (SIL) as endpoints. However, cervical cytology has only moderate clinical sensitivity (55%-65%) for detection of histopathologically confirmed 'true disease status' [18], and with low inter- or intraobserver'true correlation, even one grade of misclassification can result in highly distorted outcomes on incidence or regression, especially with varying thresholds of disease positivity. Very few studies report diagnosis of CIN by colposcopy and histopathology[20,26]. While most investigators have measured progression of cervical lesions independently, a prominent study has combined persistence and progression into one comparison group[14]. Researchers also differed in their treatment of atypical squamous cells of undetermined significance (ASCUS). Some considered a change of cytology result from low grade SIL (LSIL) to ASCUS as regression, [13] others resolved diagnoses of ASCUS with colposcopy and histology,[27] and still others left ASCUS diagnoses out of analysis[28].

Our review covers studies that evaluate the effects of HAART on incidence, progression, and regression of HPV infection and CIN lesions/ICC. We only include studies among HIV-infected women which have controlled for cervical treatment to tease out the independent effect of HAART on recurrent lesions. We present salient features of studies grouped by impact of HAART on HPV infection, on CIN, and on ICC, in Tables 2, 3, 4, 5 and 6.

**Table 2:** Summary of studies analyzing the impact of ART on HPV incidence, persistence and clearance among HIV-infected women

Author	Outcome	Follow-up	Results Comparison and reference groups	Association (95% CI), p value	Conclusion
Lillo et al 2001	HPV PCR biannually Outcome: Incidence or Persistence (same HPV genotype at enrollment and follow-up)	Median 15.4 months	Incidence: HAART vs. no therapy Persistence: HAART vs. no therapy	OR 0.28 (0.09-0.86), *p *0.02 OR 1.18, (0.37-3.77), *p *0.77	HAART protective against new HPV infections No impact of HAART on persistence
Del Mistro et al 2004	HPV PCR every 6-12 months Outcome: Persistence and Clearance (undefined)	49% of N followed for >3 years	No odds ratios discussed	--	"Prevalence and clearance of HPV not associated with HAART"
Paramsothy et al 2009	HPV PCR every 6 months for 10 visits, then annually Outcome: HPV Clearance (2 negative HPV PCR tests at consecutive visits)	Median 2 years for women on HAART; 2.7 years not on HAART	HAART vs. no HAART (adjusted for CD4+ counts) (i) in women with LSIL or HSIL (ii) in women with ASCUS on cytology (iii) in women with normal cytology	(i) HR 4.5 (95% CI: 1.2 16.3) (ii) HR 1.0 (95% CI: 0.4-2.5) (iii) HR 1.7 (95% CI: 0.9-3.1)	HAART promoted clearance of HPV infection in women with LSIL or HSIL on cytology. No impact of HAART on women with ASCUS cytology No impact of HAART on women with normal cytology

**Table 3:** Summary of studies analyzing the impact of ART on incidence of cervical disease among HIV-infected women

Author	Outcome	Follow-up	Results Comparison and reference groups	Association (95% CI), p value	Conclusion
Ellerbrock et al 2000	Histologically confirmed SIL cytology result	3-12 month period	ARV therapy vs. no ARV therapy	RR 1.0 (0.5 - 2.0), *p 0.94*	ART has no impact on incidence of SIL
Schuman et al 2003	Pap result of LSIL, HSIL	4 years (median)	HAART vs. no HAART	RR 1.2 (0.49 - 2.94), *p *0.7	HAART has no impact on incidence of SIL
Heard et al 2006	Pap change from normal to LSIL or HSIL	28 months (median)	HAART vs. no HAART	RR 0.7 (0.4 - 1.2), *p *0.15	HAART has no impact on incidence of SIL
Sirera et al 2007	Pap change from normal to LSIL or HSIL in all women on HAART	Study period 1997 - 2005	CD4+ <200 cells/mm^3 ^vs. >200 cells/mm^3 ^(All participants on HAART)	OR 0.38 (0.14 - 1.01), *p *0.05	HAART has no impact on incidence of SIL
Sirera et al 2008	Pap change from normal to LSIL or HSIL	Study period 1997 - 2006	HAART vs. no HAART	OR 1.84 (0.72 - 4.69), *p *0.20	HAART has no impact on incidence of SIL
Soncini et al 2007	Histologically confirmed CIN	11 years	HAART vs. other NRTI or no ARV therapy, adjusted for CD4+	HR 0.3 (0.13 - 0.68), *p *0.004	Only study showing HAART prevents incidence of CIN

**Table 4:** Summary of studies analyzing the impact of ART on progression of cervical disease among HIV-infected women

Author	Outcome	Follow-up	Results Comparison and reference groups	Association (95% CI), p value	Conclusion
Orlando et al 1999	Pap result lower to higher grade lesions	6 months; 114 days (median)	CD4+ <200/mm^3 ^vs. CD4+ ≥ 200/mm^3 ^(all women on HAART)	OR 2.18 (95% CI and p-value not reported)	Unclear (incomplete reporting)
Schuman et al 2003	Pap result of LSIL, HSIL	4 years (median)	HAART vs. no ART	OR 1.5 (0.90 - 2.49), *p *0.12	HAART has no impact on progression of SIL
Paramsothy et al 2009	Pap result: Normal to ASCUS, ASCUS to LSIL, LSIL to HSIL	2 years for women on HAART, 2.7 years not on HAART	HAART vs. no HAART	HR 0.7 (0.6 1.0), *p *>0.05	HAART has no impact on progression of SIL
Lillo et al 2001	Pap result: Normal to LSIL or HSIL and LSIL to HSIL	15.4 months (median)	HAART vs. no ART, adjusted for CD4+	OR 2.01 (0.44 - 9.20), *p *0.36	HAART has no impact on progression of SIL
Del Mistro et al 2004	Pap result: "persistence or worsening"	49% followed for >3 years	HAART vs. no ART	19/36 (53%) women vs. 8/16 (50%) women, *p *NR	Unclear (incomplete reporting)
Minkoff et al 2001	Pap result: Normal to ASCUS, ASCUS to LSIL, LSIL to HSIL	6 months: consecutive paired results on each participant	HAART vs. no HAART, adjusted for CD4+ and initial Pap result	OR 0.68 (0.52-0.88), *p *NR	Only study showing evidence that HAART prevents progression of SIL

**Table 5:** Summary of studies analyzing the impact of ART on regression of cervical disease among HIV-infected women

Author	Outcome	Follow-up	Results Comparison and reference groups	Association (95% CI)	Conclusion
Minkoff et al 2001	Pap result: Normal to ASCUS, ASCUS to LSIL, LSIL to HSIL	Variable follow-up	HAART vs. no HAART, adjusted for CD4+ and initial Pap result	OR 1.4 (1.04 - 1.82), *p *NR	HAART has no impact on regression of SIL
Ahdieh - Grant et al 2004	2 consecutive normal Pap smears after LSIL or HSIL	At least 7 years	Regression rate in person years; HAART vs. no HAART	12.5% (9.9 - 15.1%) vs. 0%, OR/RR not reported	Unclear (incomplete reporting)
Heard et al 1998	Pap result: SIL	12 months	Regression rate (%) in women on triple-drug HAART versus those not on HAART	35% vs. 12.5%, *p *0.001	HAART promotes regression of SIL
Heard et al 2002	High grade CIN or HSIL and low grade CIN or LSIL or normal.	17.7 months (median)	HAART vs. no HAART, adjusted for CD4+	HR 1.93 (1.14 - 3.29), *p *0.01	HAART promotes regression of SIL
Del Mistro et al 2004	Pap result: "persistence or worsening"	49% followed for >3 years	HAART vs. no ART	OR 0.36 (0.08-1.62), *p *NR	HAART has no impact on regression of SIL
Moore et al 2002	High grade CIN to lower grade CIN	10 months (median) (IQR: 8 - 14)	HAART naïve versus HAART - experienced	OR 1.9 (0.28, 12.87), *p *0.51	HAART has no impact on regression of SIL
Paramsothy et al 2009	Pap result: Normal to ASCUS, ASCUS to LSIL, LSIL to HSIL	2 - 2.7 years	HAART vs. no HAART	HR 1.3 (1.0 - 1.7), *p *>0.05	HAART has no impact on regression of SIL
Schuman et al 2003	Pap result of LSIL, HSIL	4 years (median)	HAART vs. no ART	OR 0.86 (0.50 - 1.47), *p *0.57	HAART has no impact on regression of SIL

**Table 6:** HAART and Incidence of Invasive Cervical Cancer

Author	Outcome	Design	Results Comparison and reference groups	Association measures and CI	Conclusion
Intl Collaboration on HIV and Cancer 2000	Cases of ICC in multiple population studies	Meta-analysis	1997-1999 (post-HAART) vs. 1992-1996 (pre-HAART)	Adj IR 2.1 vs. 1.1; RR 1.87 (99% CI 0.77 - 4.56)	Higher incidence post HAART years, but not stats. significant
Dorucci et al 2001	Cases of ICC in Italian HIV Seroconversion Study	Prospective cohort/time-series analysis	1996-1998 (post-HAART) vs. 1981-1995 (pre-HAART)	IR 4.9 vs. 1.5; RH of Incidence 4.25 (0.8 - 28.24)	Higher incidence post HAART years, but not stats. significant
Clifford et al 2005	Cases of ICC from Swiss Cancer Registry	AIDS-Cancer Match Study	HAART vs. no HAART	SIR 0 vs. SIR 11.4; RR not estimatable	Unclear (incomplete reporting)
Engels et al 2006	Cases of ICC in HIV/AIDS Cancer Match Study	AIDS-Cancer Match Study	1996-2002 (post-HAART) vs. 1990-1995 (pre-HAART)	SIR 5.3 vs. SIR 4.2; RR pr year: 1.04 (95% CI: 0.94-1.15)	Higher incidence post HAART years, but not stats. significant
Biggar et al 2007	Cases of ICC in HIV/AIDS Cancer Match Study	AIDS-Cancer Match Study	1996-2002 (post-HART) vs. 1990-1995 (pre-HAART)	IR 86.5 vs. 64.2; RR of Incidence 1.41 (95% CI 0.81-2.46)	Higher incidence post HAART years, but not stats. significant
Engels et al 2008	Cases of ICC in HIV/AIDS Cancer Match Study	AIDS-Cancer Match Study	1996-2002 (post-HAART) vs. 1991-1995 (pre-HAART)	SIR 2.9 vs. 3.1; RR 0.8 (95% CI 0.3 - 2.0)	Lower incidence post HAART years, but not stats. significant
Dal Maso et al 2009	Cases of ICC in Italian Cancer Registries	AIDS-Cancer Match study	1997-2004 (post-HAART) vs. 1986-1996 (pre-HAART)	SIR 41.5 vs. 51.0; RR not reported	Lower incidence in post HAART years, but conclusion unclear (incomplete reporting)

**Foot note to tables**: Abbreviations:

ASCUS = atypical squamous cells of undetermined significance

CD4+ = CD4+ T lymphocyte count (cells/mm^3^)

DHHS = U.S. Department of Health and Human Services

HAART = Highly active antiretroviral therapy

HERS = HIV Epidemiology Research Study

HIV = Human Immunodeficiency Virus

HPV = Human papillomavirus

HR = Hazard ratio

HSIL = high-grade squamous intraepithelial lesions

ICC = Invasive cervical cancer

LSIL = low-grade squamous intraepithelial lesions

NRTI = Nucleoside reverse transcriptase inhibitor

NNRTI = Non-nucleoside reverse transcriptase inhibitor

NR = Not reported

OR = Odds ratio

Pap = Papanicolaou smear

PI = Protease inhibitor

RR = Relative risk (rate ratio)

SIR = Standardized incidence ratio

WIHS = Women's Interagency HIV Study

In the sections below, we discuss the salient study findings that highlight the heterogeneity in studies and the current dearth of evidence to definitively demonstrate the impact of HAART on HPV-associated cervical neoplastic disease in HIV-infected women.

### Studies on impact of HAART and Incidence, Prevalence and Clearance of HPV infection

Studies that evaluate HPV infection have been limited, primarily because of limited availability of high quality HPV polymerase chain reaction (PCR) typing on cervical samples, while simultaneously evaluating the outcomes of cytological/colposcopic abnormalities over time among HIV-infected women seeking HAART. (Table 2) In an Italian cohort study of 201 HIV-infected women followed for up to 6 years, antiretroviral therapy regimens were not associated with increased prevalence/persistence or regression of HPV infection[14]. Another Italian prospective cohort of 168 HIV-infected women also found no independent effect of receiving HAART with progression or regression of HPV infection, although higher and increasing CD4+ T-cell counts were associated with lower rates of HPV persistence[15]. This same study reported, however lower incidence of HPV-16 and 18 infections in women receiving HAART compared to women not on any treatment or women treated only with reverse transcriptase inhibitors (RTIs)[15]. A recent analysis from the HIV Epidemiology Research Study (HERS), a longitudinal multi-centric cohort of HIV-infected or at-risk HIV-uninfected women in the US, has also found an increased rate of HPV clearance among HIV-infected women on HAART who were diagnosed with SIL[23]. (Table 2) HAART did not have an impact on HIV-infected women with normal or ASCUS Pap results.

In summary, the evidence about the impact of HAART on incidence, progression and clearance of HPV infection and lesions remains inconsistent and inconclusive. While these cohort studies are limited by their modest sample sizes in general, this limitation is especially significant when establishing impact on individual HPV types or phylogenetically similar types of HPV. It is expected that with improved techniques, expanded availability, and standardization of HPV primers that is being attempted at an international level, [29], future studies will be able to address these limitations. This will be even more important in the era of HPV vaccination targeted at high-risk HIV-infected women[30]. Yet, measurement of HPV infection is always confounded by the fact that most, if not all, detected HPV infections are transient, especially with the constantly fluctuating immunological milieu among HIV-infected women.

### Studies evaluating the impact of HAART on incidence of cervical lesions

Since the goal of early detection (screening) for cervical cancer is to target precancerous lesions, studies that measure the impact of HAART on these lesions have significant implications for informing clinical practice as well as public health guidelines. Results from a pre-protease inhibitor-era cohort of HIV-infected women from the United States did not report a protective effect of antiretroviral treatment on SIL, even after controlling for CD4+ status and HPV-DNA status[31]. In a well-characterized French cohort with a median follow-up period of 28 months, there was no independent effect of receiving antiretroviral therapy or restored immune status (evidenced by higher CD4+ T-cell counts) on incident SIL, which were significantly associated only with being in the 30-39 years age group[22]. In a retrospective analysis of a cohort from Spain of HIV-infected women with CD4+ cell counts >350 cells/mm^3 ^and with no previous SIL, there was no significant difference in SIL incidence between groups receiving versus not receiving HAART[28].

Similar findings are also reported from another Spanish cohort that indirectly assessed the impact of HAART on incidence of SIL as outcome on its effect on CD4+ T-cell counts or its effect on HIV-1 viral loads[24]. The HERS cohort results also report no decreased relative risk of incident SIL in HIV-infected women on HAART[25]. Only one prospective cohort study, following 101 HIV-infected women in Italy, has reported that being on HAART reduced SIL incidence as compared to not being on HAART, but was not able to distinguish the independent effect of HAART versus NRTI combination antiretroviral therapy on SIL incidence[20].

Thus, most published literature [22,24,25,28,32] (except one prospective cohort study [20]) suggests that being on HAART does not reduce the incidence of cervical precancerous lesions in HIV-infected women. (Table 3) While studies have been limited by the nature and duration of treatment regimens, there are other hypothesized explanations for this finding. SIL/CIN represent accumulative oncogenic changes in cells of the squamocolumnar junction of the cervix caused due to persistent HPV infections that may not be readily altered by the changing nature of immunological status induced in the short term by HAART. Additional evidence in this area will continue to accrue through new studies that will follow-up HIV-infected women (many if not all without any initial presence of SIL/CIN) while comparing differences in duration needed for development of these lesions through active and periodic detecting incident SIL/CIN lesions.

### Studies evaluating the impact of HAART on progression of cervical lesions

Studies evaluating the impact of HAART on progression of preexisting SIL have reported mixed results. (Table 4) Studies from the HERS cohort do not report an independent impact of HAART on progression of cervical lesions. A 2003 paper from HERS cohort reported that being on HAART was not an independent predictor of progression of SIL, although poorer immune status (reflected by CD4+ cell counts <200/mm^3^) was associated with significantly higher odds of progression[25]. A more recent analysis from the same cohort found that women on HAART with SIL were 30% less likely to progress, but the difference was not statistically significant[23]. The two Italian cohorts discussed above also reported no significant difference in rates of progression between those treated with HAART compared to those not receiving any treatment[14,15]. However, a 2001 paper from the other well-characterized multicentric cohort in the United States, the Woman's Interagency Health Study (WIHS), reported that HAART significantly decreased cytological progression even after controlling for CD4+ count and baseline Pap result [17] in a 6-month follow-up paired analysis (pair of consecutive visits). Cytological progression was less likely among those with lower HIV viral loads but was not associated with CD4+ status. Progression was more likely among those with persistent HPV infection.

Thus, data on impact of HAART on progression from low to higher grade SIL/CIN remains inconclusive with conflicting study results. While it is biologically possible that immune competence restored due to HAART may in fact prevent the progression, it appears more likely that progressive lesions are reflective of slow oncogenic changes due to persistent HPV infection that are possibly unaffected to the relatively short duration and typically moderate immunocompetence induced due to HAART. Comparison across studies is difficult since they differ in how they adjust for markers of immunological status of the patient (e.g. CD4+ counts, duration of being HIV-infected) as well as differences in HAART (e.g. duration of being on HAART, adherence and effectiveness of HAART).

### Studies evaluating the impact of HAART on regression of cervical lesions

A 2001 report from the WIHS study concluded that women on ART who were infected with at least 1 high-risk HPV genotype were 40% more likely to experience regression of SIL than those not receiving ART, after adjustment for CD4+ cell counts and baseline cytology status[17]. The follow-up study report from the WIHS cohort found a regression rate of 12.5 per 100 person years among HAART recipients (significantly associated with lower post-HAART CD4+ counts) compared to no regression of lesions among HIV-infected women before HAART was introduced [13] Yet, this regression rate was five times lower than that of HIV negative women. The French cohort (discussed previously) reported first in 1998 and then in 2002 that women on HAART had 2-3 times the risk of regression than in women not on HAART[27,33]. A 2004 Italian study also found that the rate of regression of LSIL was higher among HAART recipients[14]. However, the findings from the HERS cohort suggests that regression of SIL is not independently associated with HAART status. In the 2003 report, SIL decreased by 22% for every log_10 _increase in HIV viral load, but this decline was not independently related to CD4+ counts or HAART status[25]. Also, although in the recent HERS analysis, while HIV-infected women on HAART were 30% more likely to exhibit regression on their cytology results than those not on HAART, this difference did not reach statistical significance[23]. Other studies have measured impact of surgical excisive treatment on recurrence of HPV-mediated cervical lesions, and correlated it with HAART status[34,35]. However, it is not possible to tease out the independent effect of HAART in such situations and hence we have not discussed these studies in our review.

Thus, the evidence that HAART causes regression of lesions is also mixed just as the evidence about HAART on progression of lesions. In addition to the challenges in controlling for the markers of immune suppression and the type and nature of HAART, these cohort studies also differ by varying definitions of clinical endpoints of SIL/CIN, possible misclassification of results, and the small sample sizes. Moreover, in the context of regression, the immunological and virological interplay between HIV, HPV, and HAART is not particularly well-understood. The pathways by which HIV may interact with HPV are still under investigation[36]. The HIV-1 tat gene has been shown to enhance HPV early gene expression, which is important in the cell transformation and the process of SIL development [37,38]. Some studies in HIV-infected women have also shown a decrease in vaginal Langerhans' cells, which promote local cervical cellular immunity[39,40]. Other mucosal cytokine factors have also been suggested as influencing local disease manifestation in HIV/HPV coinfection[41]. There is some limited in-vitro data to suggest that some HAART drugs, particularly protease inhibitors, may have an anti-tumor effect independent of increased immunocompetence, [42], although this has not been proven in clinical studies.

### Studies evaluating the impact of HAART on rates of invasive cervical cancer

In 2000, the International Collaboration on HIV and Cancer pooled incidence estimates of ICC among all till-date prospective cohort studies in North America, Europe, and Australia in the pre-HAART and post-HAART eras. The study reports no difference in ICC incidence rates pre- or post HAART, although the pooled cases of ICC in the study were too few (36) to make definitive comparisons with the impact of HAART on other AIDS-associated cancers such d non-Hodgkin lymphoma[9]. The Italianas Kaposi's Seroconversion Study followed a prospective cohort of 483 women through pre and post-HAART eras found increased hazards of incidence of ICC in the post-HAART period but was also limited by the small sample size[10]. AIDS-Cancer Registry Matching studies have been attempted in both Europe (Italy, Switzerland) [19,21] and in the United States[11,12,16]. The data has revealed mixed evidence with some suggesting higher risk in the post-HAART era [11,16] and others the opposite[12,19]. However, none of the reported associations in these studies are statistically significant, given their low absolute numbers of incident cases of invasive cervical cancer.

It has been suggested, however, that some record linkage methodologies may have underestimated the risks of some cancers among people withAIDS[43]. Thus, it is difficult to interpret the significance of these findings that suggest little change in the numbers of ICC between the pre- and post-HAART eras. Most authors also note the difficulty in comparing incidence of ICC across time due to competing mortality from other causes in HIV-infected women. Additionally, all these studies were conducted in industrialized settings, where the greater access to frequent cytological screening and early treatment services (rather than HAART) may have actually prevented ICC among the population of HIV-infected women.

### Gaps in evidence and directions for further research

Evident in this review is that most research on the impact of HAART on cervical cancer has been conducted and reported from high-income nations while the developing world unquestionably shoulders a disproportionate burden of the morbidity and mortality associated with cervical cancer[1]. The same nations, especially those in Africa, also struggle with significant burden due to HIV/AIDS, disproportionately affecting women[44,45]. The need for clear, evidence-based screening and treatment guidelines is especially imperative in these settings as hundreds of thousands of HIV-infected women are now accessing HAART and are starting to live longer in a moderately immunocompetent state. In these settings, a clearer understanding of the impact of HAART on cervical cancer in HIV-infected women is necessary not only from an academic and scientific perspective but also from a resource allocation and program implementation point of view. Prevention research in cervical cancer is often within the context of clinical care. Recent efforts through vertical HIV/AIDS care and treatment programs such as the U.S. President's Emergency Plan for AIDS Relief (PEPFAR) have started focusing on including cervical cancer screening as an integral component of HIV/AIDS care and treatment [46-48]. Newer screening methods such as HPV testing also need to be incorporated into more sensitive screening protocols for HIV-infected women[49,50].

Clearly, a trial randomizing women to HAART versus no HAART to study impact of HAART on incidence, progression or regression of HPV infection or CIN would be unethical. Yet, opportunities for answering these questions abound. It is quite feasible to nest outcomes research studies in HIV/AIDS care and treatment settings that may allow for accumulation of data in developing evidence-based guidelines for cervical cancer prevention in HIV-infected women. Some possible approaches to develop this evidence in the context of public health implementation programs and clinical, epidemiological, or implementation research studies could include:

#### 1. Nesting observational studies within pre-existing HIV-related study cohorts

Dovetailing observational studies within pre-existing cohorts of HIV-infected women followed-up primarily for other HIV/AIDS related (non-cervical cancer) outcomes could be valuable. In many such cohort studies, especially those in middle incomes countries, data of varying degrees of completeness exists for cervical disease status (determined by cytology, visual inspection-based screening, or colposcopy/histology) and HAART status for HIV-infected women. These data can be gainfully exploited for evaluating associations between the HAART and cervical neoplasia that may have context specific significance.

#### 2. Conducting quasi-experimental studies in HAART delivery settings

In settings where HAART programs are being newly instituted, it may be possible to conduct innovative quasi-experimental studies to answer some important research questions. In these settings, a large proportion of HIV-infected women have never been screened for cervical cancer. By providing cervical cancer screening services through cost-effective and locally appropriate methods, it is possible to compare CIN disease status (baseline and follow-up) of cervical screening-naïve women who are initiating HAART newly and those who are HAART experienced for longer periods (e.g. >6 months). This design may provide valuable research opportunities without additional investments or formal randomization procedures for HAART exposure.

#### 3. Nesting studies within HIV-related randomized trials

Multiple studies are underway evaluating the utility of early versus deferred initiation of HAART among HIV-infected persons[51,52]. Such studies provide ideal experimental venues for answering questions related to the degree of impact of HAART on HPV/cervical outcomes, especially considering that by design randomization would control for differences in HAART status in the two groups of women being compared.

#### 4. Sentinel surveys

In settings with large numbers of women being treated with HAART, sentinel surveys can be conducted by documenting cervical disease status at predetermined frequencies and pre-determined sample sizes to assess changes in CIN outcomes over time. With careful data collection approaches and quality assurance measures, such sentinel sites will serve as important resources for monitoring disease trends over longer periods of time.

## Conclusion

There remain significant challenges for elucidating the impact of ART on CIN disease outcomes. As evidenced in the review of studies above, the interactions between HIV, HPV, HAART and the development of cervical neoplasia are not clearly understood. Evidence of the impact of HAART on incidence, progression and regression of CIN is largely mixed. Given the small absolute numbers of invasive cervical cancer cases in HIV-infected women in industrialized nations, the role of HAART on ICC outcomes is difficult to determine with certainty. The reasons for the wide variation in the medical literature likely reflect complex immunological or viral-immune interactions yet to be elucidated. The heterogeneity of HPV and its ubiquity in populations, the difficulty in diagnosing CIN, and its long natural history may also play a role. Certainly, the biological interactions between HIV, HPV, and HAART appear different from other cancers of viral origins common in HIV-infected persons.

Innovative approaches are needed to elucidate the impact of HAART in HIV-infected women in resource-limited settings. Given that the nature of this research is intertwined with clinical care, conduct of such epidemiology studies dovetailed within existing public health care and treatment programs for HIV-infected women can have significant patient benefits as well as provide insights to the interplay between two preventable diseases: AIDS and cervical cancer.

### Footnote to manuscript

* Significant differences exist while describing the drug treatments for HIV

(antiretroviral therapy). In this article, we use the term highly active antiretroviral therapy (HAART) broadly in place of antiretroviral therapy (ART), although but most published literature only uses the terminology HAART to describe the more effective combination regimens introduced after 1996 and those currently recommended by the World Health Organization.

## Competing interests

The authors declare that they have no competing interests.

## Authors' contributions

VS conceived the manuscript idea and developed it along with LB. LB conducted the data abstraction. The manuscript was drafted by both LB and VS and both authors read and approved the final manuscript.
